# Bioinformatic characterisation of genes encoding cell wall degrading enzymes in the *Phytophthora parasitica* genome

**DOI:** 10.1186/1471-2164-15-785

**Published:** 2014-09-11

**Authors:** Leila M Blackman, Darren P Cullerne, Adrienne R Hardham

**Affiliations:** Plant Science Division, Research School of Biology, College of Medicine, Biology and Environment, The Australian National University, Canberra, ACT 0200 Australia; CSIRO, Agriculture Flagship, Canberra ACT, 2601 Australia

**Keywords:** CAZymes, Carbohydrate binding module, Carbohydrate esterase, Cell wall degrading enzymes, Glycoside hydrolase, Polysaccharide lyase, *Phytophthora parasitica* genome

## Abstract

**Background:**

A critical aspect of plant infection by the majority of pathogens is penetration of the plant cell wall. This process requires the production and secretion of a broad spectrum of pathogen enzymes that target and degrade the many complex polysaccharides in the plant cell wall. As a necessary framework for a study of the expression of cell wall degrading enzymes (CWDEs) produced by the broad host range phytopathogen, *Phytophthora parasitica*, we have conducted an in-depth bioinformatics analysis of the entire complement of genes encoding CWDEs in this pathogen’s genome.

**Results:**

Our bioinformatic analysis indicates that 431 (2%) of the 20,825 predicted proteins encoded by the *P. parasitica* genome, are carbohydrate-active enzymes (CAZymes) involved in the degradation of cell wall polysaccharides. Of the 431 proteins, 337 contain classical N-terminal secretion signals and 67 are predicted to be targeted to the non-classical secretion pathway. Identification of CAZyme catalytic activity based on primary protein sequence is difficult, nevertheless, detailed comparisons with previously characterized enzymes has allowed us to determine likely enzyme activities and targeted substrates for many of the *P. parasitica* CWDEs. Some proteins (12%) contain more than one CAZyme module but, in most cases, multiple modules are from the same CAZyme family. Only 12 *P. parasitica* CWDEs contain both catalytically-active (glycosyl hydrolase) and non-catalytic (carbohydrate binding) modules, a situation that contrasts with that in fungal phytopathogens. Other striking differences between the complements of CWDEs in *P. parasitica* and fungal phytopathogens are seen in the CAZyme families that target cellulose, pectins or β-1,3-glucans (e.g. callose). About 25% of *P. parasitica* CAZymes are solely directed towards pectin degradation, with the majority coming from pectin lyase or carbohydrate esterase families. Fungal phytopathogens typically contain less than half the numbers of these CAZymes. The *P. parasitica* genome, like that of other Oomycetes, is rich in CAZymes that target β-1,3-glucans.

**Conclusions:**

This detailed analysis of the full complement of *P. parasitica* cell wall degrading enzymes provides a framework for an in-depth study of patterns of expression of these pathogen genes during plant infection and the induction or repression of expression by selected substrates.

**Electronic supplementary material:**

The online version of this article (doi:10.1186/1471-2164-15-785) contains supplementary material, which is available to authorized users.

## Background

The ability to penetrate the formidable physical barrier of the plant cell wall is fundamental to successful pathogen invasion of plants and is facilitated by the secretion of cell wall degrading enzymes (CWDEs) by the pathogen. These extracellular effectors degrade a wide range of complex and cross-linked polysaccharides and glycoproteins. Pathogen CWDEs function not only in plant penetration but also in the release of nutrients for pathogen use. They are important determinants of pathogenicity [e.g. NCBI: NP_521723 and NP_522144; [1]].

The intricate, interconnected molecular network that constitutes the plant cell wall is centred around three types of polysaccharides: cellulose, hemicellulose and pectin [2]. Cellulose microfibrils consist of 30 to 50 β-1,4-linked glucan chains held together by intramolecular and intermolecular hydrogen bonds to form an insoluble scaffold [3]. Cellulose microfibrils are further cross-linked by hydrogen bonds to hemicellulose molecules and both are embedded in a pectin matrix. Hemicelluloses have a backbone of β-1,4-linked glucose, xylose, mannose and, sometimes, galactose units that are substituted with different side chains whose residues may be modified by the addition of acetyl or methyl groups [4–6]. Xyloglucans are the most abundant hemicellulose and consist of four subunits containing β-1,4-linked glucan backbones substituted with α-1,6-xylosyl, β-1,2-galactosyl and α-1,2-fucosyl residues in a variety of combinations [3, 4].

The most structurally diverse group of wall polysaccharides is the pectins [7]. The three main forms of pectin are homogalacturonan (HG), rhamnogalacturonan I (RGI) and rhamnogalacturonan II (RGII). HG is the simplest and most common form of pectin in plant cell walls. It consists of chains of α-1,4-linked-D-galacturonic acid residues which are secreted in a methyl esterified form and which may also be acetylated at the O-2 and O-3 positions [4, 8]. Cross-linking of unmethylesterified HG by calcium allows close packing of the HG chains and gives pectin its gel-like properties [3]. RGI polysaccharides consist of a backbone of α-1,2-rhamnosyl and α-1,4-galacturonic acid residues [5, 9]. The rhamnosyl residues may be substituted with side chains having a diversity of lengths and compositions, including α-linked arabinose residues (arabinans) and β-1,4-galactose linked α-1,3-L-arabinose residues (arabinogalactans), with some side chains also containing L-fucose and D-glucuronic acid residues [3, 9]. α-1,4-galacturonic acid residues in the backbone may be acetylated. RGII is a highly complex polysaccharide present as dimers linked by a borate ester with backbones of at least seven α-1,4-linked galacturonic acid residues with a diversity of substitutions that are yet to be characterized [10].

Structural and biochemical properties of plant cell walls vary between dicotyledons and monocotyledons. For example, β-1,3:1,4-linked glucans are found only in grasses [6]. Plant cell walls also contain proteins and glycoproteins that may cross-link wall polysaccharides, thus strengthening the wall. They often contain glycosylphosphatidyl inositol (GPI) anchors and may function in connection of the wall with the plasma membrane. In glycoproteins, diverse carbohydrate chains are attached via the N in asparagine (*N*-linked oligosaccharides), by the O in serine/threonine (*O*-linked oligosaccharides) or by hydroxyproline residues (arabinogalactan proteins: AGPs) [11]. In *N*-linked oligosaccharides, mannose and *N*-acetylglucosamine residues form the backbone of the linked carbohydrate moiety [11]. In *O*-linked oligosaccharides, *N*-acetylgalactosamine residues form the carbohydrate backbone [12]. In AGPs, β-1,3- and β-1,6-galactose residues are joined to hydroxyproline and are substituted with many different saccharides including L-fucose, L-rhamnose and D-xylose [13].

The complex nature of cellulose, hemicellulose, pectins and glycoproteins and their interactions within the cell wall mean that the plant cell wall is a structurally diverse and effective barrier to plant pathogens [2, 14]. Typically, plant cell wall structure and composition differs in different plant tissues and cell types [2] and changes during growth and development and in response to biotic and abiotic stress [5, 15]. For example, β-1,3-glucans (callose) are often deposited at the site of pathogen invasion, creating, it is believed in at least some plant-pathogen interactions, a wall that is more resistant to pathogen penetration [15, 16].

Synthesis, modification and degradation of the complex carbohydrates that form plant cell walls require large numbers of highly specific enzymes [17]. The *Arabidopsis thaliana* genome, for example, contains 730 genes encoding proteins involved in these processes and the *Aspergillus nidulans* genome contains 224 genes for proteins specifically involved in wall degradation [18, 19]. To aid research in this field, protein motifs that confer carbohydrate catalytic activity have been classified into sequence-related families of Carbohydrate-Active enZyme (CAZyme) modules [19]. These modules are divided into six classes – glycoside hydrolases (GHs), polysaccharide lyases (PLs), carbohydrate esterases (CEs), glycosyltransferases (GTs), auxiliary activities (AAs) and non-catalytic, carbohydrate-binding modules (CBMs) [20–22]. The activity of proteins within these classes has been annotated according to sequence homologies, protein folding and known enzyme activities [20]. Many CAZyme proteins contain a number of different modules, allowing them to target specific or divergent substrates [21, 23–26].

Oomycetes, including *Phytophthora* species, are major plant pathogens worldwide. Like fungal phytopathogens, Oomycete species produce a wide range of cytoplasmic and extracellular effector proteins that facilitate their successful infection of host plants [27, 28]. Over recent years, a number of Oomycete genomes have been sequenced, providing a wealth of information for studies of Oomycete effectors and pathogenicity mechanisms. Analyses of genomes from *P. sojae, P. infestans*, *P. ramorum*, *Pseudoperonospora cubensis* and *Pythium* species have catalogued CWDEs that contain CAZyme modules in these organisms [29–32], however, the regulation of CAZyme production and the role of individual CWDEs during plant infection remains largely unknown. The bioinformatic study reported in the present paper builds on the identification of CAZymes in these other Oomycetes to generate a comprehensive analysis of the complement of CWDEs in the broad host range pathogen, *P. parasitica*. Sequence and motif characterizations have been used to explore likely functions of individual *P. parasitica* CAZyme proteins. The study provides the framework for future studies of the expression of *P. parasitica* CWDEs during plant infection.

## Methods

### Identification of *P. parasitica*CWDEs

Predicted proteins were downloaded from the *P. parasitica* INRA-310 Sequencing Project [33] and screened for carbohydrate-active modules using Carbohydrate-active enzyme ANnotation [dbCAN, [34, 35]. CAZyme module annotation by this program uses E-value, alignment length and coverage, with an E-value of <1e-5 for alignments of >80 amino acids and an E-value of <1e-3 for alignments of <80 amino acids. Proteins containing GT modules were identified but not included in further analysis. To eliminate proteins identified by dbCAN but that were not really CAZymes, proteins with CAZyme motifs with a dbCAN E-value > e-10 were individually examined using MyHits [36], Prosite [37, 38] and BLASTp of non-redundant protein sequences in National Center for Biotechnology Information (NCBI) [39]. Proteins for which the alignment coverage was less than 0.5, were also individually examined. Putative CWDEs were those identified as containing GH, PL, CE, CBM and AA modules that are known to be associated with the degradation of carbohydrates associated with the cell wall. To identify CWDEs that might have been missed by dbCAN, additional searches including keyword and Pfam [40, 41] domain searches and tBLASTn analysis [42] with characterised proteins listed on the CAZy site [43] were conducted. When there was ambiguity in the identification of a CWDE, CAZymes Analysis Toolkit (CAT) [44, 45] was employed. Some proteins were also checked against data in the partially-completed Version 3 of the *P. parasitica* genome [46]. Transcripts identified by dbCAN that were predicted to have two unrelated functions were considered to be genome assembly errors and were not included in subsequent analysis. When a protein contained a CBM as well as a GH motif, the protein was classified according to the GH catalytic activity.

Because CAZyme annotation is based on sequence similarity rather than substrate specificity, enzymes that act on different substrates are placed within the same family and, conversely, enzymes acting on the same substrate are found in different families [26]. Where possible, the putative function of *P. parasitica* CAZymes has been deduced by comparisons with characterised proteins using BLAST analysis and identification of key catalytic residues. Classical signal peptides for secretion (SPs) were identified using SignalP 4.1 [47, 48] and proteins targeted to the non-classical secretion pathway identified using SecretomeP 1.0f [49, 50]. Pathogen CWDEs need to be secreted in order to function in wall degradation, however, putative CWDEs lacking an *in silico*-recognised SP were retained in the analysis because this *in silico* analysis is not 100% reliable [51, 52]. For proteins where an SP was not detected, the 5' end of the *P. parasitica* INRA-310 genomic sequence was manually examined for nearby alternative start codons. Transmembrane domains (TMD) were identified using Phobius [53] and TMHMM [54, 55]. Potential GPI anchors were identified with PredGPI [56, 57].

When a gene appeared to be truncated, the missing sequence data were sought in the immediate upstream or downstream region within the supercontig. *Phytophthora* EST data in NCBI and, in some cases, the orthologous coding region in other *P. parasitica* isolates (P1569, P1976, P10297 and CJ01A1) [33] were used to correct the INRA-310 isolate gene models. Where data from one of these other *P. parasitica* isolates has been used to modify an INRA-310 isolate gene sequence, the name of the source isolate is shown as a superscript above the INRA-310 protein ID number from the Broad Institute sequencing project [33]. Protein similarities and identities were determined by multiple sequence and pairwise alignments of full-length proteins, omitting the SP if present [58, 59]. Proteins were considered to be homologs if they shared 25% or more identity with an alignment length greater >80 amino acids [60].

## Results and discussion

### Identification of CAZyme modules in *P. parasitica*predicted proteins

A total of 750 CAZyme modules were identified in 671 of the 20,825 predicted proteins of *P. parasitica* using dbCAN (Additional file 1: Table S1). Of these, 240 proteins were eliminated from further analysis because they either contained a GT module (151 proteins) or were CAZymes that would function intracellularly. BLAST searches identified 19 additional putative CWDEs that had carbohydrate-active domains recognised by Pfam27.0 and other protein motif analysis programmes but not by dbCAN. The total of 431 putative CWDE proteins were classified into 14 CBM, 34 GH, eight CE, three PL and four AA families (Tables 1, 2, 3, Additional file 2: Table S2). Two CBM modules (CBM25 and CBM43) occurred only in conjunction with a GH module and were included in the GH family. Analysis of these 431 *P. parasitica* proteins showed that 327 contained a classical N-terminal SP, 67 contained non-classical secretion sequences, 70 contained a TMD and 31 contained a GPI anchor sequence.

**Table 1:** ***P. parasitica*** **proteins containing glycosyl hydrolase (GH) modules**

CAZyme family	Substrate	Enzyme activity	EC number	Number	References
GH1	cellulose	β-glucosidase	3.2.1.21	17	18, 64, 66, 67,
	hemicellulose (xyloglucans)	exo-β-1,4-glucanase	3.2.1.74		72-74
	pectin (RGI)	β-galactosidase	3.2.1.23		
		β-mannosidase	3.2.1.25		
GH2	hemicellulose (mannans)	β-mannosidase	3.2.1.25	1	64
	glycoproteins (mannans)				
GH3	cellulose	β-glucosidase	3.2.1.21	25	64, 97
	pectin (RGI)	exo-β-1,4-glucosidase	3.2.1.74		
	hemicellulose (xyloglucans)	xylan β-1,4-xylosidase	3.2.1.37		
	AGPs	glucan β-1,3-glucosidase	3.2.1.58		
		α-L-arabinofuranosidase	3.2.1.55		
GH5	cellulose	endo-β-1,4-glucanase	3.2.1.4	22	23, 64, 76, 97
	hemicellulose (xylans)	β-1,4-cellobiosidase	3.2.1.91		
	hemicellulose (galactomannans)	endo-β-1,4-xylanase	3.2.1.8		
	β-1,3-glucans	endo-β-1,4-mannosidase	3.2.1.78		
		glucan β-1,3-glucosidase	3.2.1.58		
GH5/CBM43	β-1,3-glucans	glucan β-1,3-glucosidase	3.2.1.58	3	23, 78
GH6	cellulose	endo-β-1,4-glucanase	3.2.1.4	7	18, 79
		cellobiohydrolase	3.2.1.91		
GH7	cellulose	endo-β-1,4-glucanase	3.2.1.4	5	77, 80
		reducing end-acting cellobiohydrolases	3.2.1.176		
GH10	hemicellulose (xylans)	endo-β-1,4-β-xylanase	3.2.1.8	4	97
GH12	cellulose	endo-β-1,4-glucanase	3.2.1.4	15	18, 66, 81, 82
	hemicellulose (xyloglucans)	xyloglucan endo-β-1,4-glucanase	3.2.1.151		
GH13	starch	α-amylase	3.2.1.1	1	141
		α-glucosidase	3.2.1.20		
GH13/CBM25	starch	α-amylase	3.2.1.1	1	141
		α-glucosidase	3.2.1.20		
GH16	hemicellulose (xyloglucans)	xyloglucan endo-β-1,4-glucanase	3.2.1.151	25	94-96
	β-1,3-glucans	β-1,3-glucosidase	3.2.1.39		
GH17	β-1,3-glucans	endo-β-1,3-glucosidase	3.2.1.39	17	39
GH17/CBM13	β-1,3-glucans	endo-β-1,3-glucosidase	3.2.1.39	3	22, 88
GH18	*N*-linked oligosaccharides	endo-β-N-acetylglucosaminidase	3.2.1.96	3	64, 132
GH19	*N*-linked oligosaccharides	endo-β-N-acetylglucosaminidase	3.2.1.14	2	64
GH28	pectin (HG)	polygalacturonase	3.2.1.15	18	111, 112
GH30	cellulose	β-glucosidase	3.2.1.21	17	18, 64, 66, 68,
	hemicellulose (xyloglucans)	endo-β-1,4-xylanase	3.2.1.8		75, 99
	pectin (RGI)	xylan β-1,4-xylosidase	3.2.1.37		
	AGPs	endo-β-1,6-galactanase	3.2.1.164		
		β-1,6-glucanase	3.2.1.75		
GH30/CBM13	hemicellulose (xyloglucans)	xylan β-1,4-xylosidase	3.2.1.37	4	22, 88
GH31	starch	α-glucosidase	3.2.1.20	9	18, 143, 144
	hemicellulose (xyloglucans)	α-xylosidase	3.2.1.177		
GH31/CBM25	starch	α-glucosidase	3.2.1.20	1	18, 92
GH32	sucrose	invertase	3.2.1.26	5	146, 147
GH35	hemicellulose (xyloglucans)	β-galactosidase	3.2.1.23	1	115
	pectin (HG); AGPs	exo-β-1,4-galactanase	3.2.1.-		
GH37	trehalose (α,α-1,1-glucans)	α,α-trehalase	3.2.1.28	2	43
GH38	*N*-linked oligosaccharides	α-mannosidase	3.2.1.24	1	136
GH43	hemicellulose (xylans)	α-L-arabinofuranosidase	3.2.1.55	7	97, 98
	pectin (RGI)				
	AGP				
GH47	*N*-linked oligosaccharides	α-mannosidase	3.2.1.113	5	136
GH53	pectin (RGI)	endo-β-1,4-galactanase	3.2.1.89	6	116
GH54	pectin (RGI)	α-L-arabinofuranosidase	3.2.1.55	1	97, 117, 118
GH63	*N*-linked oligosaccharides	processing α-glucosidase	3.2.1.106	1	137
		α-1,3-glucosidase	3.2.1.84		
		α-glucosidase	3.2.1.20		
GH72	β-1,3-glucans	β-1,3-glucanosyl-transglycosylase	2.4.1.-	14	130, 131
GH78	pectin (RGI)	α-L-rhamnosidase	3.2.1.40	4	113
GH81	β-1,3-glucans	endo-β-1,3-glucanase	3.2.1.39	16	127, 128
GH85	AGPs	endo-β-N-acetylglucosaminidase	3.2.1.96	1	138
GH89	*N*-linked oligosaccharides	α-N-acetylglucosaminidase	3.2.1.50	2	43, 139
GH105	pectin (RGI)	unsaturated rhamnogalacturonyl hydrolase	3.2.1.172	1	114
GH109	*O*-linked oligosaccharides	α-N-acetylgalactosaminidase	3.2.1.49	7	43
GH123	*O*-linked oligosaccharides	glycosphingolipid β-N-acetylgalactosaminidase	3.2.1.-	1	43
GH131	cellulose (β-1,4-glucans) hemicellulose (β-1,4-glucans)	exo-β-1,3/1,6-glucanase with endo-β-1,4-glucanase	3.2.1.-	5	83
Total				280	

Potential substrates, likely enzyme activities, EC number and the number of family members are shown. The accession numbers of proteins in each GH category are tabulated in Additional file 2. Assessments of likely substrates and enzyme activities were based on information provided in the cited references. HG = homogalacturonan; AGP = arabinogalactan protein; RGI = rhamnogalacturonan I.

**Table 2:** ***P. parasitica*** **proteins containing auxiliary activity (AA) and carbohydrate-binding (CBM) modules**

CAZyme family	Substrate	Enzyme activity	EC number	Number	References
AA7	cellobiose	glucooligosaccharide oxidase	1.1.3.-	5	21, 87
	chitin/glycoproteins	chitooligosaccharide oxidase			
AA8	cellulose	iron reductase domain	na	3	21
AA9	cellulose	copper-dependent monooxygenase	1.-	1	21, 84-86
AA10	cellulose	copper-dependent monooxygenase	1.-	4	21, 24, 84, 85
Total				13	
CBM1	cellulose			17	22, 30, 90, 93
CBM9	hemicellulose (xylans)			1	92
CBM13	hemicellulose (xylans)			4	22, 88
CBM18	chitin/glycoproteins			1	133
CBM20/CBM47	starch/fucose			1	78, 142
CBM32	galactose, PGA and β-galactosyl-β-1,4-GlcNAc			1	126
CBM37	hemicellulose (xylans)			1	
	cellulose				78
CBM38	inulin-binding			1	43
CBM40	sialidases			2	78
CBM47	fucose binding			3	78
CBM50	chitin/glycoproteins			3	133-135
CBM57	domains associated with glycosidases			1	43
CBM63	cellulose			12	22
Total				48	

Potential substrates, likely enzyme activity, EC number and the number of family members are shown. References used to determine the potential activities of each family are shown. CBMs do not have catalytic activity. The details of each protein in each family are found in Additional file 2. PGA = polygalacturonic acid; GlcNAc = N-acetylglucosamine; na = not available.

**Table 3:** ***P. parasitica*** **proteins containing carbohydrate esterase (CE) and polysaccharide lyase (PL) modules**

CAZyme family	Substrate	Enzyme activity	EC number	Number	References
CE1	hemicellulose	feruloyl esterase	3.1.1.73	3	43, 45, 93, 101
CE2	hemicellulose	acetyl xylan esterase	3.1.1.72	1	100, 102
CE3	hemicellulose	acetyl xylan esterase	3.1.1.72	1	100, 103
CE4	hemicellulose	acetyl xylan esterase	3.1.1.72	2	100, 104, 105
	*N*-linked oligosaccharides	peptidoglycan GlcNAc deacetylase	3.5.1.-		
CE5	hemicellulose	acetyl xylan esterase	3.1.1.72	4	100, 107-109
		cutinase	3.1.1.74		
CE8	pectin (HG)	pectin methylesterase	3.1.1.11	15	18, 123
CE12	pectin (HG, RGI)	pectin and RGI acetylesterase	3.1.1.-	14	18, 125
		acetyl xylan esterase	3.1.1.72		
CE13	pectin (HG)	pectin acetylesterase	3.1.1.-	6	124
Total				46	
PL1	pectin (HG)	pectate lyase	4.2.2.2	21	119-121
		exo-pectate lyase	4.2.2.9		
		pectin lyase	4.2.2.10		
PL3	pectin (HG, RGI)	pectate lyase	4.2.2.2	17	119, 122
PL4	pectin (RGI)	rhamnogalacturonan lyase	4.2.2.-	6	119, 122
Total				44	

Potential substrates, likely enzyme activity, EC number, the number of family members and references used to identify putative activities are shown. Proteins accession numbers and other characteristics are tabulated in Additional file 2. HG = homogalacturonan, RGI = rhamnogalacturonan I; GlcNAc = N-acetylglucosamine.

*P. parasitica* proteins with multiple CAZyme modules were relatively rare, with only 50 of the 431 proteins (i.e., 11.6%) having more than one module. In 37 cases, the protein contained two or more copies of the same CAZyme module. These included 29 proteins with two GH16, CBM1 or PL1 modules and three proteins with either three or six CBM13 modules. In the other 13 cases, the protein contained two CAZyme modules of different types. In plants and fungi, CE and GH modules are often accompanied by CBM modules [61, 62]. As an example, only 4.3% of *P. parasitica* GH proteins also contained a CBM module whereas 15.5% of GH proteins from *A. thaliana* also have a CBM module [61].

CAZyme classification relies upon amino acid sequence but since tertiary structure and substrate binding is dependent upon primary structure, the substrate of many CWDEs can be inferred from the primary sequence data. However, it is possible for slight differences in protein folding arising from small variations in amino acid sequence to change the targetted substrate [63]. Where possible, sequence homology with characterized CAZymes has been used to group predicted CWDEs according to the substrate(s) they degrade.

### CAZymes involved in cellulose degradation

The β-1,4-linked glucose residues that form cellulose can be degraded by enzymes that fall into four broad categories: endo- and exo-β-1,4-glucanases [17, 18, 64], cellobiohydrolases [64, 65] and β-glucosidases [66]. There are also AA proteins that assist in cellulose breakdown through direct or indirect enzyme activity [21], and CBMs that bind to cellulose and act to concentrate enzyme activities. In some cases, CBMs may mediate non-catalytic disruption of the crystalline structure of cellulose, thereby facilitating degradation via catalytic modules [22]. In *P. parasitica*, CAZyme families containing exo-β-1,4-glucanases include GH1 and GH3 while families containing endo-β-1,4-glucanases include GH5, GH6, GH7, GH12 and GH131. Cellobiohydrolases are found in GH5, GH6 and GH7 and β-glucosidases occur in GH1, GH3 and GH30 families. Cellulose is not the sole substrate for many of the CAZymes in these families and hemicelluloses and pectins are often also targetted. *P. parasitica* AA proteins consist of two types of monooxygenases that cause direct oxidative cleavage (AA9 and AA10), and one family of reductases (AA8) that produce hydroxyl radicals resulting in the non-enzymatic breakdown of cellulose chains [21]. The genome also contains two CBM families that are specific for cellulose [22].

#### Glycosyl hydrolases

**Figure 1:**
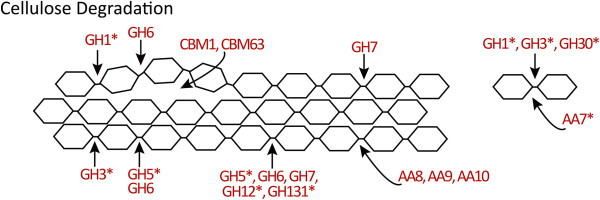
**Diagrammatic representation of cellulose showing putative target sites for predicted** ***P. parasitica*** **CAZymes during cellulose degradation.** The assigned enzymatic specificities are based on *P. parasitica* sequence homologies to characterised CWDEs. Asterisks indicate enzymes that are likely to act on more than one substrate.

GH1, GH3 and GH30 families include enzymes that target disaccharides and the terminal non-reducing end of a range of polysaccharides (Figure 1) [18, 64, 66–68]. Fungal phytopathogens typically have small GH1 families [69–71] but in *P. parasitica* there are 17 GH1 proteins, many of which contain conserved residues important for β-glucosidase activity [67]. It was not possible to determine specific enzyme activities of the *P. parasitica* GH1 proteins based on sequence homologies with characterized enzymes but 11 [PPTG_12005, 12006, 12007, 12009, 12010, 12011, 12051, 12052, 12102, 19353 and 19709] contained a TMD in the C-terminal region and overall share 29% amino acid identity (Additional file 2: Table S2). The implied membrane-anchorage may be indicative of a role of these enzymes in the modification of *Phytophthora* rather than of host cell walls. For example, *A. thaliana* contains plasma membrane located β-glucosidases from both GH1 and GH3 families and they are thought to act in the production and modification of the plant cell wall [72]. TMDs have been found in some fungal GH9 β-glucanases from the basidiomycete, *Phanerochaete chrysosporiumi*, and in plant proteins containing GH9 modules that are involved in plant cell wall modification [73, 74].

**Figure 2:**
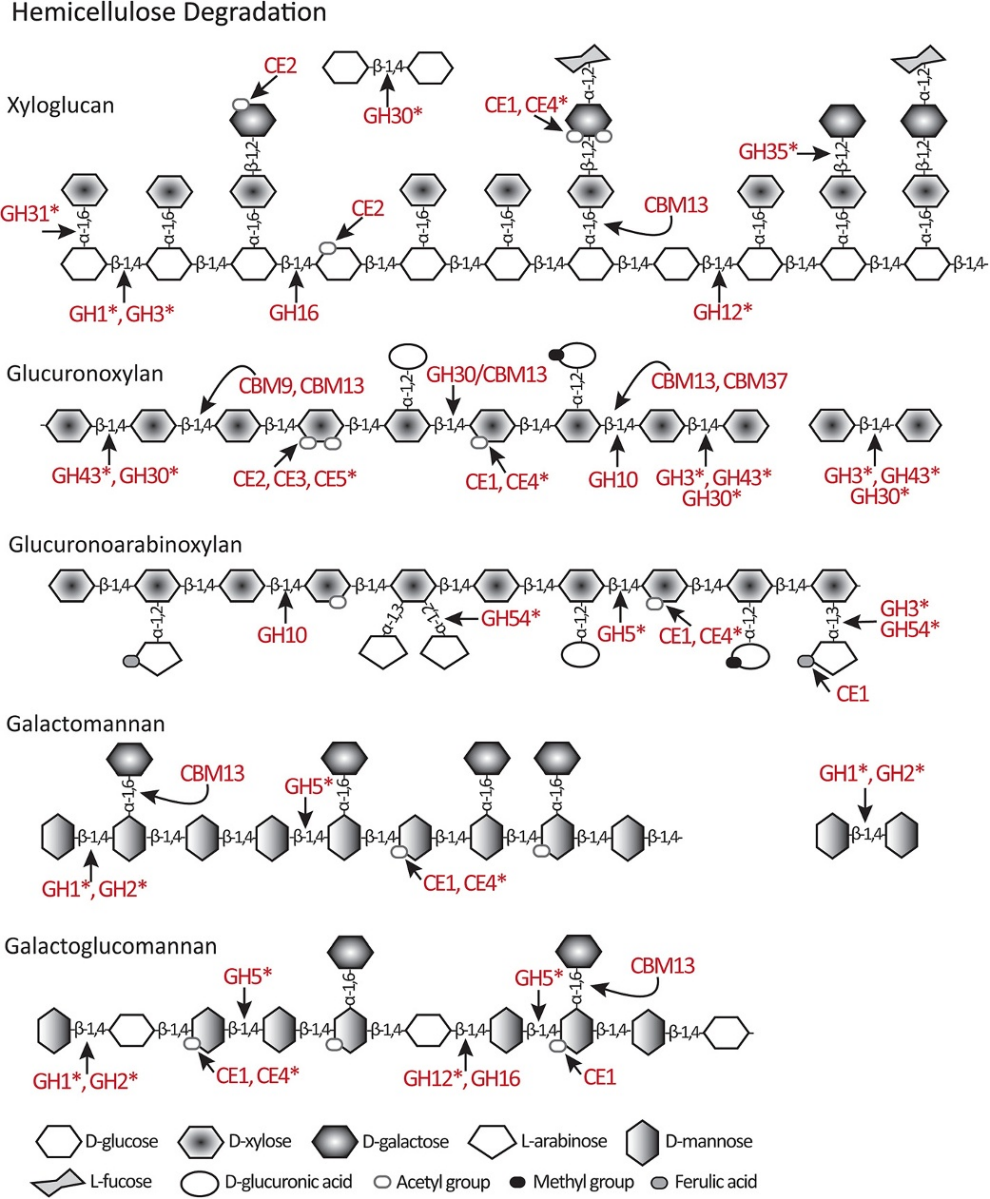
**Diagrammatic representations of a range of hemicellulosic polysaccharides showing putative target sites for predicted** ***P. parasitica*** **CAZymes during hemicellulose degradation.** The assigned enzymatic specificities are based on *P. parasitica* sequence homologies to characterised CWDEs. Asterisks indicate enzymes that are likely to act on more than one substrate.

GH3 is the third largest CAZyme family in *P. parasitica* with 25 members (Table 1). Many fungal phytopathogens also have large GH3 families [69–71]. GH3 proteins act on disaccharides and on the non-reducing end of a range of polysaccharides to release single residues such as glucose or xylose (Figures 1 and 2) [64]. GH3 proteins may also be involved in the degradation and processing of AGPs [64]. An alignment of 17 similar-sized *P. parasitica* GH3 proteins showed that they had little overall homology (3%) but these proteins could be grouped into four subfamilies, with members of each subfamily sharing 24, 38, 46 or 55% amino acid identity. Comparisons with enzymes with known activities did not provide any clues as to the specific substrate of each GH3 subfamily.

Fungal GH30 families contain only a few members whereas most Oomycetes sequenced to date have large GH30 families [31, 69–71]. *P. parasitica* contains 21 predicted GH30 proteins. Sequence alignments allowed the *P. parasitica* GH30 family to be divided into three subfamilies. The sequences of 10 *P. parasitica* proteins [PPTG_03369, 07665, 09210, 09211, 09212, 09214, 09215, 09216, 09217 and 14237] in one subfamily were similar to an 85 kDa β-glucosidase/xylosidase from *P. infestans* designated BGX1, with one protein, PPTG_09215, sharing 81% identity [NCBI: AAK19754; [75]]. BGX1 releases terminal β-glucose residues, and to a lesser extent β-xylose residues, from artificial substrates, suggesting that this group of *P. parasitica* GH30 proteins is likely to be involved in the release of glucose residues from β-1,4-linked glucans in both cellulose and xyloglucans. The other two subfamilies of *P. parasitica* GH30 proteins act on hemicellulose and AGPs and will be dealt with in a later section.

The GH5 CAZyme family is diverse. It includes endo-β-1,4-glucanases, β-1,4-cellobiosidases, endo-β-1,4-mannanases, exo-β-1,3-glucosidases, endo-β-1,6-galactanases and β-1,6-glucanases and is one of the largest GH families in many symbiotic, biotrophic, hemibiotrophic and necrotrophic fungi. For example, the biotroph *Puccinia triticina* contains 28 GH5 CAZymes [20% of all GH CAZymes; [71]], the hemibiotroph *Colletotrichum higginsianum* contains 22 GH5 CAZymes [6% of all GH CAZymes; [70]] and the necrotroph *Stagonospora nodorum* contains 18 [6% of all GH CAZymes; [70]]. In *P. parasitica*, GH5 is one of the two largest CWDE families. The 25 GH5 proteins (Table 1) represent 9% of *P. parasitica* GH CAZymes; similar numbers of GH5 proteins are found in other characterised Oomycete species [31, 76]. All *P. parasitica* GH5 proteins are potentially secreted, three contain GPI anchors [PPTG_01140, 01151 and 03220] and 11 contain TMDs (Additional file 2: Table S2). Because the main microfibrillar component in cell walls of *Phytophthora* and other Oomycete species is cellulose [77], rather than chitin as in fungi, these membrane-anchored endoglucanases may be involved in pathogen wall modification as proposed for fungal GH5_9 proteins [23]. Ten GH5 proteins appear to be endo-β-1,4-glucanases, including eight [PPTG_03844, 03845, 03846, 03847, 04558, 05786, 05787 and 05788] that are homologous to GH5_20 subfamily proteins from other *Phytophthora* species [23]. *P. parastica* GH5_20 proteins contain a TMD at the predicted N-terminus. Three GH5 proteins also contain a CBM43 module (Additional file 2: Table S2) and are similar to previously identified proteins in the Stamenophile-specific GH5_33 subfamily of exo-β-1,3-glucanases [e.g. NCBI: CBJ27054; [23]]. While not all members of this subfamily have a CBM43 module, its presence would provide affinity for β-1,3-glucans [78]. Six other GH5 proteins were identified as potential β-1,3-glucosidases [PPTG_01483, 11518, 13105, 16240, 16244 and 16244] but had limited homology to the GH5_33 proteins.

Endo-β-1,4-glucanase activity also occurs in GH6, GH7, GH12 and GH131 families (Figure 1). All seven *P. parasitica* GH6, five GH7, 14 full-length GH12 and all five GH131 proteins have features for non-classical secretion or signal peptides directing secretion. The GH6 proteins share many conserved residues, including those found in dual-action endoglucanase-cellobiohydrolases [79]. GH6 enzymes act at the non-reducing end of the polysaccharide chain. The five *P. parasitica* GH7 proteins share 29% amino acid identity and contain the ExDxxE motif typical of the GH7 family [65]. The GH7 family includes cellobiohydrolases that release the disaccharide cellobiose from the reducing end of cellulose chains, endo-β-1,4-glucanases and β-1,3-1,4 glucanases. Sequence alignment showed that the GH7 proteins could be split into two subfamilies but specific functions could not be assigned to individual GH7 proteins, although two [PPTG_07012 and 07017] sharing 71% amino acid identity showed sequence similarities with β-1,3:1,4 glucanases from *Bispora* sp. [e. g. NCBI: ACT53749, [80]] and the other three showed more similarity to endoglucanases from *Trichoderma reesei* [65]. Using sequence data from two orthologous genes [PPTG_11504^P10297^ and 16275^P10297^] from another *P. parasitica* isolate to predict full-length versions of truncated proteins in the INRA-310 isolate, the 14 *P. parasitica* GH12 proteins were shown to contain the six GH12 consensus boxes described in Goedegebuur *et al.* [81]. *P. parasitica* GH12 proteins shared 19-100% pairwise amino acid identity and this sequence diversity is similar to that seen in fungal GH12 endoglucanases [81]. While the GH12 family includes proteins with endo-β-1,4-glucanase, xyloglucanase, β-1,3-1,4-glucanase and xyloglucan endotransglycosylase activity, sequence analysis suggests that the GH12 proteins from *Phytophthora* will hydrolyse β-1,4-glucan chains in cellulose or xyloglucans. Master and coworkers [82] have shown that *P. sojae* and *P. ramorum* GH12 proteins are closely related to a xyloglucan-specific endo-β-1,4-glucanase GH12 from *Aspergillus. P. parasitica* contains five proteins from the recently identified GH131 family. The few members of this family that been characterised have a number of enzyme activities and can act on β-1,4-linked glucans as well as terminal β-1,3- and β-1,6-linked glucans [83].

#### Auxiliary redox enzymes

Recent research has led to the addition of the AA family of CAZymes to the original five GH, PL, CE, CBM and GT families within the CAZy database [21]. Currently there are 11 AA families listed in the CAZy database [43], some of which are polysaccharide oxygenases which are ostensibly involved in lignin breakdown but which also target cellulose in lignocellulose [21]. *P. parasitica* has 13 potentially secreted proteins from four AA families. Enzymes with AA8 modules are iron reductases, providing reactive oxygen species to assist in cellulose chain degradation [21]. Three *P. parasitica* proteins contain an AA8 module and share 42% identity (Table 2 and Additional file 2: Table S2). Although AA8 proteins typically also contain a C-terminal CBM1 module, thought to bring the enzyme and its reaction product close to the cellulose molecule [21], this motif did not occur in any *P. parasitica* AA8 proteins.

AA9 (formerly classified as GH61 proteins) and AA10 (formerly CBM33) families contain copper-dependent monooxygenases [84]. Both AA9 and AA10 enzymes directly target cellulose, oxidatively cleaving the glucose chains within the cellulose microfibrils [21, 85]. AA9 enzymes are thought to oxidize the C1, C4 or C6 carbons in the glucose rings of the cellulose chains [86]. The action of AA9 and AA10 enzymes makes the cellulose more susceptible to attack by other CAZymes. AA9 proteins have been identified in a number of fungal genomes, with biotrophs containing only a few and hemibiotrophs and necrotrophs containing large AA9 families. *C. higginsianum*, for example, has 25 AA9 CAZymes [70]. To date, AA10 proteins have been predominantly found in bacteria and less commonly in eukaryotes [24]. The single AA9 and four AA10 copper-dependent monooxygenases in *P. parasitica* (Table 2 and Additional file 2: Table S2) have a high degree of similarity, sharing 42-61% pairwise identity. This sequence similarity suggests that the four genes may have arisen from a single progenitor gene through gene duplication. Examination of the *P. parasitica* scaffolds revealed that one AA10 and the sole AA9 gene are adjacent, a feature consistent with relatively recent gene duplication. *P. parasitica* also contains five secreted oxidases from the AA7 family. This family targets mono- and disaccharides and acts on a wide variety of substrates including cellobiose and α-1,4-linked glycopyranosyl residues [87].

#### Cellulose-associated CBMs

CBMs are usually auxiliary domains that occur within proteins in conjunction with other CAZyme modules [78]. It is believed that the function of CBM modules is to bring the CAZyme into close and sustained proximity to the substrate of the catalytic CAZyme modules in the enzyme, thereby increasing the catalytic efficiency of the enzyme [22, 88]. There are over 60 families of CBMs and they have been classified into three Types (A-C) according to the nature of their substrates (crystalline polysaccharides, soluble polysaccharides, or soluble mono-, di- or tri-saccharides, respectively) [22, 88].

The *P. parasitica* genome contains 29 genes encoding proteins that have CBMs that bind to cellulose (Table 2 and Additional file 2: Table S2). All contain signals for secretion with two containing GPI anchors (PPTG_04643 and 10047). Seventeen proteins belong to the CBM1 family and 12 to the CBM63 family. Both CBM1 and CBM63 are Type A CBM families that bind to crystalline cellulose [22, 88, 89].

Of the 17 *P. parasitica* CBM1 proteins, eight were identified by dbCAN analysis (Additional file 1: Table S1) and the other nine by BLAST searches and confirmed as CBM1 proteins by CAT analysis (Additional file 2: Table S2). Pairwise comparisons show that they share little sequence similarity but most appear to contain at least one copy of a domain similar to previously identified fungal cellulose binding domains [90]. The cellulose-binding and lectin-like activity of one *P. parasitica* CBM1 protein has been demonstrated experimentally [PPTG_13482; CBEL, [90]]. CBEL is one of five proteins (the other four being PPTG_05833, 06045, 07987 and 19721) that have two CBM1 domains. Although only five CBM1 proteins have been recognised in the genome of *P. infestans* [30], the much larger number of CBM1 proteins in *P. parasitica* is similar to that found in hemibiotrophic and necrotrophic fungi [30, 70, 71].

The 12 *P. parasitica* CBM63 proteins share 15-76% pairwise identity. They also contain amino acid signature sequences diagnostic of expansin-like proteins which function in the regulation of plant cell wall expansion [91]. Six of the CBM63 proteins [PPTG_18384, 18386, 18395, 18397, 19415 and 19485] also have a single TMD situated at the C-terminus and one [PPTG_19415] has two CBM63 domains. Unlike CBM1 proteins, the CBM63 family in *P. parasitica* is much larger than those found in biotrophic, hemibiotrophic and necrotrophic fungal pathogens [70, 71].

One of the interesting aspects of the cellulose-targeting CBM-containing proteins in *P. parasitica* is the fact that the CBM1 and CBM63 modules are not accompanied by catalytic modules. This is an unusual situation, only infrequently observed in CAZyme proteins [22, 78, 92]. Published reports on the CWDE complement of fungal plant pathogens have not focused on the modular structure of specific CAZyme proteins. However, an analysis of the 20 CBM1-containing proteins in *C. graminicola* revealed only a single protein containing a CBM1 and no other module [93].

### Hemicellulose degrading families

Hemicelluloses are complex heterogeneous polysaccharides defined by their solubility properties and include xyloglucans, xylans and mannans [6, 18]. Xyloglucans are the most abundant form of hemicellulose in non-graminaceous plants. They have a backbone of β-1,4-linked glucose subunits, up to 75% of which may be branched with an α-1,6-xylose residue (Figure 2). The side branches may be further elaborated by β-1,2-galactose and α-1,2-fucose substitutions. Xylans have a backbone of β-1,4-linked xylose subunits and can exist as simple unbranched chains or can contain many different side chains. In glucuronoxylans these branches are α-1,2-linked glucuronic acid subunits and in glucuronoarabinoxylans, the branches are arabinose or glucuronic acid subunits (Figure 2). Mannans possess a backbone of β-1,4-linked mannose residues and may include occasional glucose residues to form glucomannans. Backbone subunits may bear side chains of α-1,6-linked galactose residues forming galactomannans and galactoglucomannans (Figure 2). Hemicelluloses are often acetylated and sometimes linked by esterification to feruloyl or coumaroyl residues [18]. Hemicelluloses are degraded by CAZymes that digest the xyloglucan, xylan or mannan backbones and that cleave the diversity of substitutions. Activity for the degradation of hemicellulose has been described in at least 33 GH families and nine CE families [43]. In *P. parasitica* the degradation of hemicellulose potentially involves members of 12 GH and five CE families as well as non-catalytic CBMs.

#### Hemicellulose-targeting GHs

Cleavage of the xyloglucan backbone is achieved by the action of β-1,4-endoglucanases and β-1,4-glucosidases which occur in a number of GH families (Figure 2). In *P. parasitica*, the GH1, GH3, GH12 and GH30 families include proteins that act on xyloglucan backbones [18, 66, 68]. Breakage of the β-1,4-linked glucans can also be achieved by xyloglucan-specific endo-β-1,4-glucanases from the diverse GH16 family [65]. The GH16 family members also act on β-1,3-galactans in AGPs, β-1,3:1,4-glucans and β-1,3-glucans [94, 95]. Of the 25 *P. parasitica* proteins containing a GH16 module, 23 show a high degree of similarity and all but two [PPTG_09772 and 09773] contain the GH16 signature catalytic motif ExDxxE thought to be critical for xyloglucanase, galactanase and β-1,3-glucanase activity [65]; none contain the motif ExDxE found in GH16 β-1,3-1,4-glucanases [95, 96]. Most proteins from this family contain two GH16 modules and are predicted to be secreted and/or contain a C terminal TMD (Additional file 2: Table S2). None of the *P. parasitica* GH16 proteins share significant homology to known endo-β-1,3-galactanases [95]. The other two GH16 proteins lacking the ExDxxE motif [PPTG_03558 and 16550] show similarity to β-1,3-glucanases and are discussed in the section on β-1,3-glucan degradation.

**Figure 3:**
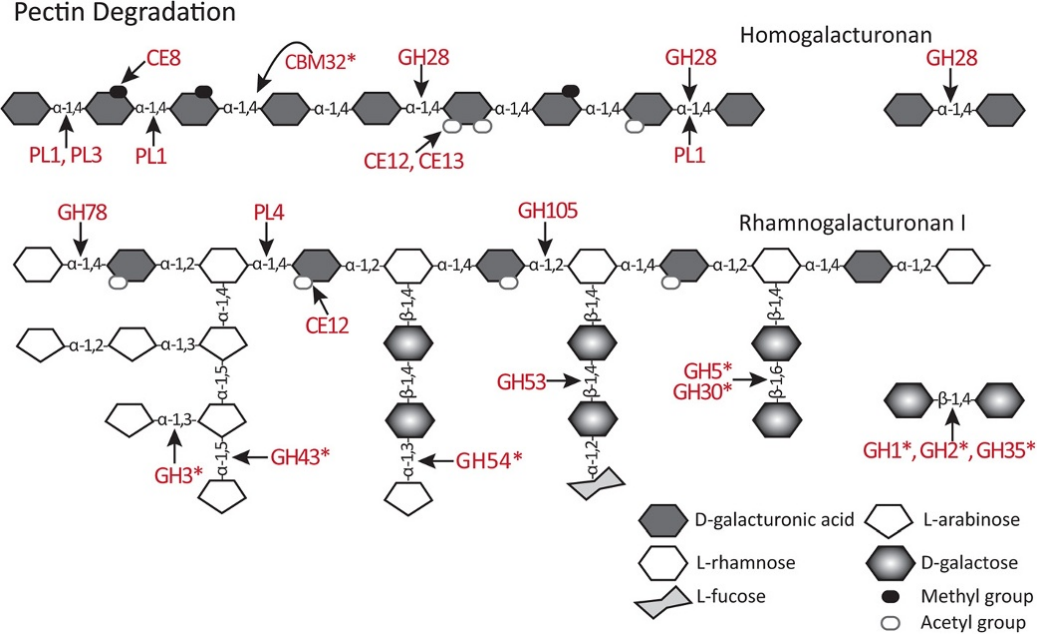
**Diagrammatic representation of two pectic polysaccharides showing putative target sites for predicted** ***P. parasitica*** **CAZymes during pectin degradation.** The assigned enzymatic specificities are based on *P. parasitica* sequence homologies to characterised CWDEs. Asterisks indicate enzymes that are likely to act on more than one substrate.

**Figure 4:**
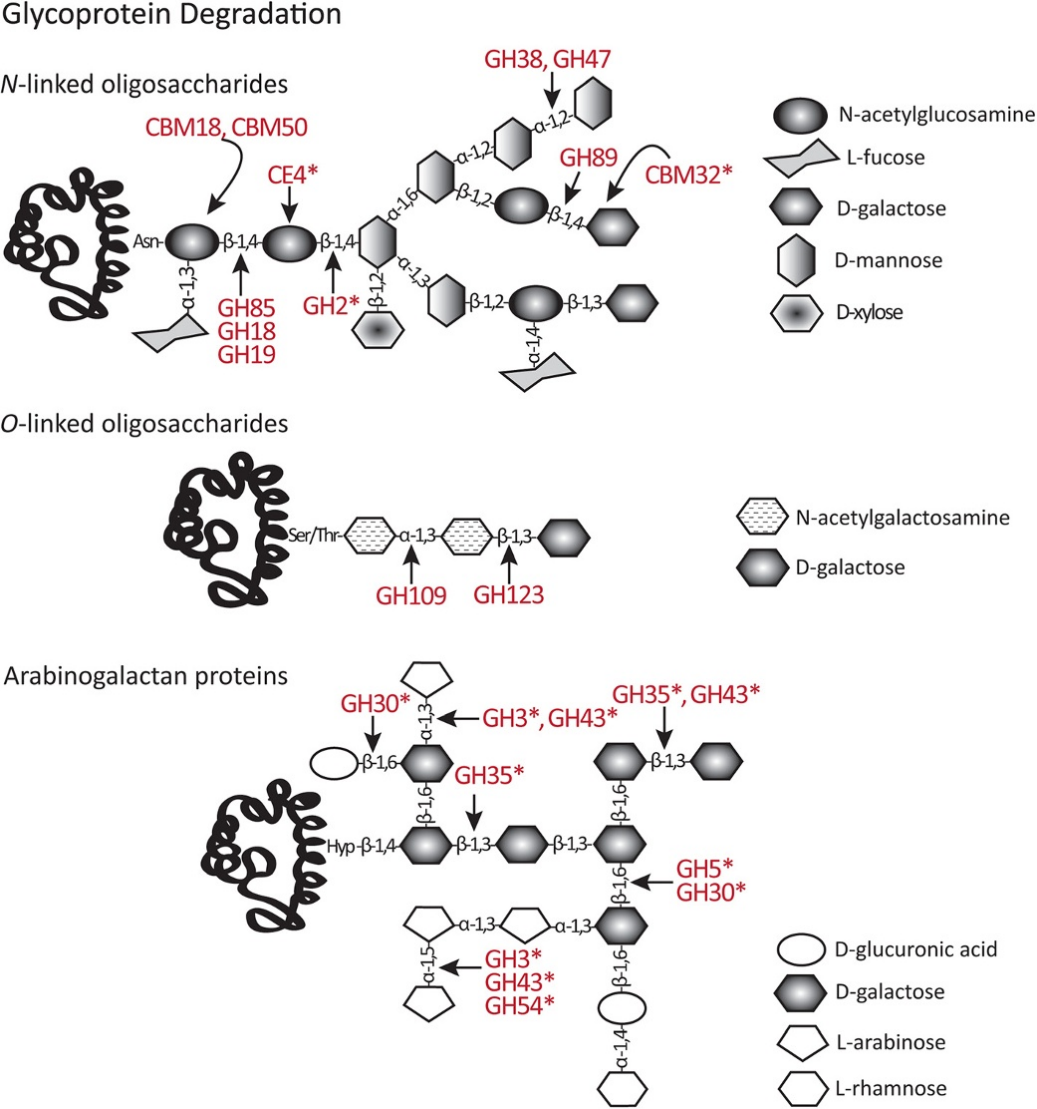
**Diagrammatic representation of three different types of glycoproteins showing putative target sites for predicted** ***P. parasitica*** **CAZymes during their degradation.** The assigned enzymatic specificities are based on *P. parasitica* sequence homologies to characterised CWDEs. Asterisks indicate enzymes that are likely to act on more than one substrate.

The β-1,4-linked xylose residues in the backbone of xylans, glucuronoxylans and glucuronoarabinoxylans are generally cleaved by endo-β-1,4-xylanases found in GH5 and GH10, and by exo-acting β-1,4-xylanases (β-1,4-xylosidases) from GH3, GH30, GH43 and GH54 families [68, 97]. The *P. parasitica* genome encodes no xylan-specific GH5 proteins but does contain genes for four putative endo-β-1,4-xylanases from the GH10 family (Additional file 2: Table S2). Three of the four have an SP and share 45% amino acid identity, but the full sequence of the fourth protein could not be resolved (Additional file 1: Table S1, PPTG_17240). Other xylanases belong to one of the GH3 family subgroups, previously described in the section on cellulose-targeting hydrolases. This group contains four proteins [PPTG_05809, 14386, 14613 and 14623] sharing 31% pairwise identity, and includes a protein annotated as a β-xylosidase [PPTG_14386]. This subgroup may thus be involved in the release of xylose residues from the end of xylans, glucuronoarabinoxylans and glucuronoxylans. Xylanases and xylosidases are also found in the GH43 family (Figure 2). In *P. parasitica* there are seven GH43 proteins and the six full-length proteins share between 56% and 88% pairwise identity [PPTG_15710, 15711, 15714, 15715, 17405 and 17407^CJ01A1^]. However, these proteins share little homology to GH43 proteins involved in xyloglucan degradation [98]. Proteins in the GH43 family can also degrade α-1,3- and α-1,5-linked arabinose-containing side chains (e.g. arabinogalactans) of hemicellulose, pectins and AGPs (Figures 2, 3, 4), and β-1,3-galactan linkages in AGPs (Figure 4). The *P. parasitica* GH43 proteins have considerable similarity to other uncharacterised GH43 arabinan endo-α-1,5-arabinosidases such as those from *A. niger* and *Myceliophthora thermophile* [e.g. NCBI: XM_001393400, XM_003663619]. Interestingly the expression of one predicted GH43 [PPTG_17407] is induced by arabinogalactan suggesting that the closely related GH43s from *P. parasitica* may be involved in the degradation of side chains and not the xylan backbone of unsubstituted xylans (G Zhang, L Sofoulis, LM Blackman and AR Hardham, unpublished results). Xylosidases are also found in the GH54 family which is described in the pectinase section.

The GH30 family, in addition to the cellulose acting β-glucosidase/xylosidases described earlier, also contains proteins that can release xylose from glucuronoarabinoxylans and act on hemicellulose side chains such as β-1,6-glucanases and β-fucosidases, and endo-β-1,6-galactanases and β-glucuronidases that degrade AGPs and pectins [43]. One *P. parasitica* group of GH30 contains five proteins [PPTG_08507, 08508, 08509, 08511 and 14259] with the four full-length proteins sharing an overall 50% amino acid identity. Members of this subfamily contain CBM13 domains (Additional file 2: Table 2), which have been shown in other organisms to associate with xylanases [22, 88]. Another group of *P. parasitica* GH30s contains six proteins [PPTG_14859, 14860, 15283, 16010 16065 and 18120], with five full-length proteins having up to 44% identity with a secreted endo-β-1,6-galactanase from *C. graminicola* [NCBI: EFQ35891]. This sequence similarity suggests that members of this subfamily of *P. parasitica* GH30 proteins, like their fungal counterparts [99], may act on arabinose- and galactose-containing side chains (arabinogalactans) found in pectins (Figure 3) and AGPs (Figure 4).

Putative *P. parasitica* enzymes with predicted activity against mannan-based hemicelluloses are found in the GH1, GH2 and GH5 families. Two proteins from the *P. parasitica* GH5 family have been annotated as endo-β-1,4-mannosidases [PPTG_03499 and 18368] and these share more similarity to characterised mannosidases [e.g. NCBI: AAS19695 from the bacteria *Cellvibrio mixtus*] than to other *P. parasitica* GH5s. A single GH2 protein containing an SP [PPTG_12094] was identified and this protein had more residues in common with a β-mannosidase from *A. nidulans* [NCBI: ABF50864] than to a β-galactosidase from *Xanthomonas campestris* pv. *campestris* [NCBI: AAP86765]. β-galactosidases that have the potential to act at the terminal non-reducing end xyloglucan side chains can also be found in GH1, GH3 and GH35, and are described later. It is possible that the *P. parasitica* GH2 may also be involved in the degradation of *N*-linked oligosaccharides (Figure 4) by cleaving the β-1,4-linkage between mannose and N-acetylglucosamine [64].

#### Hemicellulose-targeting CEs

The formation of cross-links between hemicelluloses and cellulose is moderated by the presence of acetyl, methyl and phenolic groups on backbone and side chain residues in the hemicellulose molecule [3]. Galactose residues in the backbones of xylans, glucuronoarabinoxylans and glucuronoxylans, and in the side chains of xyloglucans, galactomannans and galactoglucomannans can be acetylated and glucuronic acid residues can be methylated. Ferulic acid and coumaric acid can be attached to arabinose residues in side chains in glucuronoxylans and glucuronoarabinoxylans (Figure 2). These modifications can be removed by CEs [100]. There are currently eight known CE families with acetyl xylan esterase activity (families CE1-7 and CE16) and the *P. parasitica* genome contains members of five of these CE families (Table 3).

The CE1 family includes acetyl xylan esterase and feruloyl esterase, as well as intracellular esterases, such as S-formylglutathione hydrolase [43]. In *P. parasitica,* 34 putative CE1 proteins were identified by dbCAN with E-values between 1.4e-4 and 7.10e-48 (Additional file 1: Table S1) and another four were found by homology searches of the *P. parasitica* genome. However, none of the 38 proteins had domains or lengths consistent with CE1 proteins as indicated by CAT analysis. Currently, 96% of the 3,042 CE1 proteins listed in the CAZy database [43] are from bacteria, indicating that eukaryotes contain few CE1 proteins. Three *P. parasitica* proteins, sharing 37% identity, contain the Pfam domain PF07519 for tannase and feruloyl esterase [PPTG_00806, 06868 and 19565^P1569^], but only one of these was identified as a CE1 protein by dbCAN [PPTG_06868]. These three proteins share 20-22% amino acid residues with a characterized feruloyl esterase from *A. niger* [NCBI: CAC83933, [101]]. Interestingly, of the eight proteins annotated as tannase and feruloyl esterases in *C. graminicola*, only one has a CE1 domain [93].

A small number of putative acetyl xylan esterases from CE2, CE3, CE4 and CE5 families were identified in the *P. parasitica* genome. CE2 proteins have a preference for acetyl groups on the carbon at the C6-position of the xylose ring while CE3 esterases remove acetyl groups from a number of carbons [100]. Single acetyl xylan esterases were identified from the CE2 and CE3 families in *P. parasitica*. Fungal pathogens studied to date tend to have small CE2 families but large CE3 families [70, 71]. The *P. parasitica* CE2 protein did not have a classical or a non-classical SP but did have 40% identity with a putative CE2 from *Albugo laibachii* [NCBI: CCA14218] and 20% identity with a characterised CE2 from *Neocallimastix patriciarum* [NCBI: AAB69091 partial protein, [102]]. Homologs to the single potentially secreted *P. parasitica* CE3 protein were identified in EST libraries of mating cultures of *P. infestans* [NCBI: CV939145, [103]] and mycelia of *P. capsici* [NCBI: FG042841 and FG042841]. Genes encoding two potentially secreted CE4 proteins were found in the *P. parasitica* genome [PPTG_01441 and 12926] but both had little homology to characterised CE4 proteins [104]. PPTG_12926 contained an N-terminal TMD but PPTG_01441 did not, suggesting that these enzymes serve different functions. CE4 proteins have broad specificity (Figures 2 and 4), acting on acetylated residues in xyloglucans and xylans and on *N*-acetylglucosamine in chitin and *N*-linked oligosaccharides [105, 106].

*P. parasitica* contains four CE5 proteins. CE5 acetyl xylan esterases act predominantly on acetyl groups on the C2-position of xylose residues such as those in glucuronoxylans (Figure 2) [100, 107]. After using EST data to correct an apparent annotation error for one of these genes [PPTG_19214], all four *P. parasitica* CE5 proteins are predicted to be secreted. Three proteins [PPTG_07182, 19214 and 19215] shared 60% amino acid identity and had homology to characterised acetyl xylan esterases [NCBI: ADZ98863, [107] and cutinases [NCBI: AAA33334, [108]. However, despite the homology to cutinases, none of these three proteins contains the cutinase consensus sequence [G-H/Y-S-X-G, [109]. On the other hand, the fourth *P. parasitica* CE5 [PPTG_08907] protein, which is annotated as a lipase, does contain the cutinase consensus sequence. Two predicted CE7 proteins [PPTG_03864 and 08082] with dbCAN values of 6.80e-05 and 9.80e-12 were identified (Additional file 1: Table S1). Given that all characterized CE7 enzymes function in the cytoplasm, it seems unlikely that these two *P. parasitica* proteins are involved in the degradation of cell wall components [100].

#### Hemicellulose-targeting CBMs

Three families of CBMs with putative hemicellulose binding properties, CBM9, CBM13 and CBM37, were identified in *P. parasitica* (Table 2). In other organisms, CBM9 modules often accompany modules conveying endo-β-1,4-xylanase activity [92] but the one *P. parasitica* CBM9 protein lacks this or any other CAZyme module. CBM13 modules often occur in multi-domain proteins that preferentially target β-1,4-linked xylans but can bind to other polysaccharides [22, 88]. Four of the 10 *P. parasitica* proteins containing CBM13 modules also contain a GH30 module and are annotated as glycosphingolipid acting glucosylceramidases [PPTG_08507, 08508, 08509 and 08511]. The presence of the CBM13 modules may indicate a role for these proteins in the degradation of xylans rather than of glycosphingolipids. Three proteins [PPTG_ 09700^P10297^, 09701^P1569^, and 18983] contain a CBM13 and a GH17 module, suggesting that these may act on β-1,3-glucans. The other four CBM13 proteins contain from one [PPTG_16933], three [PPTG_09699 and 20351] to six [PPTG_15107] copies of the CBM13 module. One *P. parasitica* protein containing a CBM37 domain was identified [PPTG_01092]. CBM37 domains have been shown to bind to a number of substrates including xylan and cellulose [78]. However PPTG_01092 is predicted to have a molecular weight of over 600 kDa and contains other domains not found in CWDEs. This protein was thus not analysed further.

### Pectin degrading families

The pectin matrix of plant cell walls is constructed using three main highly complex polysaccharides, HG, RGI and RGII [9]. HG consists of unsubstituted chains of α-1,4-linked-galacturonic acid residues (Figure 3). RGI has a backbone of alternating α-1,2-rhamnosyl and α-1,4-galacturonic acid residues with different side chains substituted onto the rhamnose residues (Figure 3). The side chains include α-1,5-linked arabinans, which are in turn substituted with α-1,3-arabinans, and arabinogalactans, which consist of β-1,4-galactose substituted with α-1,3-arabinans [9]. Residues in HG and RGI can be methyl esterified or acetylated [4]. The highly complex RGIIs are dimers of seven to nine α-1,4-linked galacturonic acid residues. These backbones are highly substituted with oligosaccharides that include 20 different linkages and 12 different saccharides, forming a complex polysaccharide that is highly resistant to degradation [110]. The CAZy database includes 28 CAZyme families (18 GHs, three CEs and seven PLs) involved in the degradation of pectins. The *P. parasitica* genome includes genes in 18 of these families (12 GHs, three CEs and three PLs).

#### Pectin-targeting GHs

The HG chain of α-1,4-linked galacturonic acid residues is cleaved by GH28 endo-polygalacturonases. The GH28 family also contains exo-polygalacturonosidases and enzymes acting on the backbone of α-1,2 linked galacturonic acid and rhamnose residues in RGI. The *P. parasitica* genome contains 18 genes encoding secreted proteins from the GH28 family. All 18 proteins share a high degree of similarity, especially in the C-terminal half. Alignment of 16 full-length GH28 proteins showed they share 30% identity. Functional studies of eight *P. parasitica* GH28 proteins have demonstrated that they are endo-polygalacturonases [111, 112]. In *A. niger*, the sequences of the endo-polygalacturonases are very different from those of exo-polygalacturonosidases and rhamnogalacturonases [NCBI: ABD61567, ABD61568, CAK41025, A2QK83, ABD61564 and CAA41693] suggesting that the 18 *P. parasitica* GH28 proteins all have endo-polygalacturonase activity.

In *P. parasitica,* proteins in two other GH families are likely to be involved in the degradation of the RGI backbone, namely GH78 and GH105. GH78 enzymes specifically cleave the terminal non-reducing end α-1,2-rhamnose [113] while GH105 proteins are endo-acting enzymes that cleave the α-1,2 bond between the galacturonan and rhamnose residues [114]. *P. parasitica* has four putative GH78 enzymes, three of which are very similar (68% identity; PPTG_00925, 00925 and 00922^P1269^), and one GH105.

Nine GH families, GH1, GH2, GH3, GH5, GH30, GH35, GH43, GH53 and GH54, include proteins that have the potential to degrade RGI side chains. Rhamnose residues in the RGI backbone are substituted with side chains of β-1,4-linked galactan, α-1,3- and α-1,5-linked arabinan or branched type I arabinogalactans with small amounts of fucosyl, glucosyluronic acid and methyl β-glucosyluronic acid [4]. Potentially, the GH1, GH2, GH5 and GH30 families, described earlier, and GH35 (in which *P. parasitica* has one secreted protein), contain β-galactosidases that act on the terminal non-reducing end β-1,4-linked galactose residues in xyloglucan (Figure 2) and RGI side chains (Figure 3) and some also act on the β-1,3- and β-1,6-linked galactans of AGPs (Figure 4) [64, 115]. Unfortunately, sequence analysis has not helped determine the substrate specificity of the *P. parasitica* proteins in these five families.

*P. parasitica* has proteins in four CAZyme families, GH3, GH43, GH53 and GH54, that include enzymes that target α-1,3- and α-1,5-linked arabinan or branched type I arabinogalactans. GH3 and GH43 have been described earlier. GH53 enzymes specifically cleave the β-1,4-linked galactose residues in the arabinogalactan type I side chain of RGI. There are six *P. parasitica* GH53 proteins, of which two predicted proteins [PPTG_19165 and 19166] may be misannotated because scaffold analysis suggests that they form a single protein. The four full-length proteins share 46% amino acid identity and contain two catalytic residues typically found in endo-β-1,4-galactosidases [116]. Enzymes in the GH54 family with α-L-arabinofuranosidase activity cleave the terminal α-1,3- or α-1,5- linked arabinose residues in glucuronoarabinoxylans (Figure 2) and in arabinogalactan side chains of RGI (Figure 3) and AGPs (Figure 4). There is a single secreted GH54 in *P. parasitica* and it differs from fungal GH54 proteins in that it does not contain a xylan specific CBM42 module [117, 118]. This suggests that the *P. parasitica* GH54 protein, while sharing significant homology to the GH54 module from fungi [e.g. NCBI: BAG80559 from *Fusarium oxysporum* and NCBI: XP_003711856 from *Magnaporthe oryzae*], does not act on hemicellulose.

While an accurate estimation of the number of GH proteins involved in pectin degradation is difficult, analysis of fungal and Oomycete genomes suggests that hemibiotrophs and necrotrophs contain more pectin-degrading enzymes than biotrophs [31, 69–71]. However, the CAZyme families from which the pectin degrading GH proteins come differ between fungi and Oomycetes. Fungal pectinases occur in the GH51 and GH62 families but these two CAZyme families are not represented in the Oomycetes. Both fungi and *P. parasitica* have pectinases in GH1, GH2, GH30, GH43 and GH53 families but proteins in GH1, GH30 and GH53 families are more abundant in Oomycetes than in fungi whereas proteins in GH2 and GH43 families are more abundant in fungi. Bacteria also have GH4, GH42, GH50 and GH59 pectinases but these families are not represented in fungi or Oomycetes [43].

#### Polysaccharide lyases

CAZyme activities that degrade pectins via β-elimination are grouped in 22 PL families [119]. The *P. parasitica* genome contains 44 PLs and these occur in the PL1, PL3 and PL4 families (Table 3). All are predicted to be secreted and none contain TMDs. Both PL1 and PL3 families contain pectate lyases that cleave bonds linking the α-1,4-galacturonan residues to the HG backbone at the non-reducing end. PL1 also contains enzymes that target unesterified α-1,4-galacturonan residues at the reducing end (exo-pectate lyase) and esterified α-1,4-galacturonan residues at the non-reducing end (pectin lyase) of HG. PL4 proteins act on the RGI backbone, breaking the α-1,4 glycosidic bonds between the alternating L-rhamnose and D-galacturonic acid residues. Alignment of *P. parasitica* full-length PL1 proteins showed that they have a number of conserved residues, with pairwise identity ranging from 21% to 100%. Two PL1 proteins from *P. capsici,* Pcpel1 and Pcpel2 [NCBI: FJ213434, FJ213435] have been identified as pectate lyases, having activity against unesterified polygalacturonic acid (PGA) [120, 121]. Pcpel2 has 74-90% amino acid identity with three *P. parasitica* PL1s [PPTG_12901, 12902 and 20388] while Pcpel1 has 87% identity with one *P. parasitica* PL1 [PPTG_18908], suggesting that these *P. parasitica* proteins will act on unesterified HG. The *P. parasitica* genome contains 17 putative PL3 proteins that show a high degree of homology in their N terminal half. There are six predicted *P. parasitica* PL4 proteins but misannotation may be splitting one protein into two [PPTG_05070 and 05071]. The four that are full-length share 51-83% amino acid identity and contain the highly conserved regions thought to be responsible for binding to deacetylated RGI [122].

Although the number of CAZyme families that contain PL enzymes is similar in the Oomycete and fungal phytopathogens that have been examined, the numbers of PL proteins in *P. parasitica* and other *Phytophthora* species is more than twice that in necrotropic *Pythium* species [31] and considerably larger than in fungi [69–71]. A similar situation is seen in the pectin degrading CE families.

#### Pectin-targeting CEs

The three *P. parasitica* CE families that are not involved in hemicellulose degradation contain enzymes that are predicted to remove the methyl and acetyl groups from pectins (Table 3). The single activity of CE8 proteins is to remove methyl groups from the α-1,4-galacturonic acid residues in HG [18]. CE12 proteins remove the acetyl group from galacturonic acid residues in HG and RGI, and CE13 proteins remove the acetyl group from HG only (Figure 3). *P. parasitica* has 15 proteins in the CE8 family (Additional file 2: Table S2). Alignments of these proteins showed that they have 28% amino acid identity overall and 54-100% identity in pairwise comparisons. *P. infestans* and *P. sojae* have 11 and 19 CE8 proteins, respectively. By contrast, *Pythium* species examined to date have no CE8 proteins [31]. De-esterification by CE8 proteins is thought to be essential for subsequent degradation of HG by GH28 proteins [123].

There are 14 CE12 and six CE13 proteins in *P. parasitica*, making these families larger in *P. parasitica* than in fungal plant pathogens. Only a few fungal phytopathogens contain any CE13 proteins although they do occur in plants [69–71, 124]. The sequence of the 14 *P. parasitica* CE12 proteins is highly variable (only 8% amino acid identity) but all contain RGI acetyl esterase signature amino acid residues [125]. Sequence analysis revealed 18-74% pairwise amino acid identity, and an N-terminal TMD in another five proteins, and all are predicted to be secreted. Together, these data suggest there may be some functional differences within the *P. parasitica* CE12 family. The six CE13 proteins contain classical secretion signals and share between 38-68% amino acid identity in pairwise alignments. One CE13 protein [PPTG_19428] contains a predicted GPI anchor so it may have a different function to that of the other five CE13 enzymes.

#### Pectin-targeting CBMs

One protein containing CBM32 was identified in *P. parasitica*. CBM32 modules can interact with PGA and can be associated with *N*-acetylglucosaminidase modules [126]. CBM32 modules are found in many fungal species but, as in *P. parasitica*, are present in relatively low numbers of proteins.

### β-1,3-glucan degradation

*P. parasitica* contains three GH families, GH16, GH17 and GH81, that include proteins with predicted endo-β-1,3-glucanase activity. Of the 25 proteins in the GH16 family, 23 are predicted to act on hemicellulose or β-1,3-glucans (described earlier). Of the two remaining GH16 proteins, PPTG_16550 has homology to TOS1-like proteins [e. g. NCBI: EMR65012 from *Eutypa lata* and NCBI: EJT42179 from *Saccharomyces kudriavzevii*]. TOS1 proteins have some similarity to known β-1,3-glucanases and mutation of the *TOS1* gene from *S. cerevisiae* results in altered glycogen levels and wall modification [96]. The other GH16 protein [PPTG_03558] has a dbCAN value of 1.7e-05 and has some homology to PPTG_16550 and to non-CAZyme carboxypeptidases. There are 20 proteins in the GH17 family in *P. parasitica*, three of which [PPTG_18983, 09700^P10297^ and 09701^P1569^] also contain a CBM13 module. All but one of the full-length proteins are predicted to be secreted with two also containing a TMD at the C-terminus (Additional file 2: Table S2). The GH17 proteins lack the ExDxxE and EESTSED signature sequences found in GH16 and GH81 β-1,3-glucanases [127, 128]. All full-length proteins, while sharing little overall similarity, could be divided into subgroups and a NCBI BLASTp analysis showed that all contained a COG5309 domain found in exo-β-1,3-glucanases [39]. The GH81 family contains 16 proteins identified as endo-β-1,3-glucanases. An alignment of the GH81 proteins [including PPTG_13594^P10297^, 19888^P10297^ and 19889^P10297^] showed high sequence conservation in some regions and the presence of the EESTSED endo-β-1,3-glucanase signature motif [127]. GH17 and GH81 but not GH16 families are considerably larger in *Phytophthora* species than in other phytopathogens [31, 70, 71], a feature that may indicate a role other than degradation of the plant cell wall. These enzymes could, for example, be involved in the modification of β-1,3-glucans in *Phytophthora* walls [77, 129].

A fourth CAZyme family whose members may be involved in remodeling β-1,3-glucans via hydrolase and transferase activity is GH72 [69, 130, 131]. *P. parasitica* has 14 GH72 proteins, a number at least twice that in fungal species examined to date [70, 71]. The overall similarity between *P. parasitica* GH72 proteins is low, although five proteins share 24% amino acid identity and contain a GPI anchor, a feature typically seen in GH72 proteins from other organisms [131].

### Putative chitinases

Chitin is a polymer of *N*-acetyl-glucosamine residues and constitutes the main microfibrillar component of fungal cell walls and the exoskeletons of arthropods and insects. Chitinases act on the *N*-acetyl-β-1,4-glucosaminide linkage in both chitin and glycoproteins [64, 132]. *P. parasitica* contains proteins from two GH families that include members with chitinase activity. Three proteins belong to the GH18 family, members of which have chitinase or endo-*N*-acetyl-β-1,4-glucosaminidase activity, or even inhibit xylanase activity [132]. The two full-length *P. parasitica* GH18 proteins share only 12% amino acid identity and show similarity to mammalian, but not to plant chitinases. However, all three proteins contain regions with homology to the GH10 and GH11 binding sites in xylanase inhibitors such as XIP-1 from *Triticum aestivum* [NCBI: CAD19479]. The function of these GH18 proteins is thus unclear. GH19 family proteins characterised to date have chitinase, β-*N*-acetylglucosaminidase or lysozyme activity [64]. The two *P. parasitica* GH19 proteins are almost identical, and have sequence homology with plant chitinases. They have, for example, 27-29% pairwise amino acid identity to a chitinase from *Medicago sativa* [NCBI: ABX90065].

The *P. parasitica* genome also encodes proteins with two CBM modules (CBM18 and CBM50) that confer chitin binding [133, 134]. One *P. parasitica* protein has two CBM18 modules and an SP. In other organisms, CBM18 modules often accompany GH18 modules in chitinases [133] but this was not the case in *P. parasitica*. Three proteins had either one [PPTG_05472] or three [PPTG_09231 and 09232] CBM50 modules and two are predicted to be secreted by the non-classical pathway (Additional file 2: Table S2). PPTG_05472 was considerably larger than the other two CBM50 proteins and also contained two fibronectin type III domains, which are associated with mammalian extracellular proteins, and a LysM motif, which is known to bind peptidoglycans and chitin [135]. Homologs of this CBM50 protein are restricted to the Stramenopiles.

### Degradation of other *N*-acetylated polysaccharides and glycoproteins

The *P. parasitica* genome encodes proteins from 12 GH families that include enzymes involved in cleavage of non-chitinaceous *N*-acetylated polysaccharides and glycoproteins, some of which have been described in other sections (Figure 4). There is one representative in the GH38 family of putative glycoprotein-acting α-mannosidases [136]. There are five GH47 proteins, four of which are predicted to be secreted (Additional file 2: Table S2). GH47 proteins are associated with modification or degradation of glycoproteins containing α-1,2-linked mannose residues [136]. Two of the *P. parasitica* GH47 proteins have 33% identity [PPTG_01016 and 16418] but the other three are disimilar. One protein belonging to the GH63 family of α-glucosidases is predicted to be secreted, contained an N-terminal TMD and the three catalytic residues typical of α-glucosidases involved in *N*-glycan processing [137].

Proteins in the GH85 family are predicted to have endo-β-*N*-acetylglucosaminidase activity, to act on Asn-linked glycopeptides and to cleave between mannose and *N*-acetylglucosamine [138]. *P. parasitica* contains one GH85 protein which lacks an SP and two exo-α-D-*N*-acetylglucosaminidases with an SP in the GH89 family. In *Clostridium perfringens*, proteins from this relatively uncharacterised family act on α-*N*-acetylglucosamine-β-1,4-D-galactose residues [139]. In plants, these residues are found in inositol-glycosphingolipids [140]. Seven *P. parasitica* proteins with conserved domains typical of oxidoreductase (Pfam01408 and Pfam02894) grouped within the GH109 family which is associated with α-*N*-acetylgalactosaminidase activity [43]. One *P. parasitica* protein [PPTG_03206] had a GH123 module associated with glycosphingolipid β-*N*-acetylgalactosaminidase activity and thought to act on terminal α-linked *N*-acetylgalactosamine units from *O*-glycoproteins. The *P. parasitica* GH123 protein lacked an SP but contained a motif whose score was only slightly below the threshold for non-classical secretion as determined by SecrotomeP.

### Degradation of starch

Starch molecules contain α-1,4-linked glucans with α-1,6 branch points. The α-1,4-linked glucan chains are cleaved by endo-acting amylase or by α-glucosidase acting on the terminal, non-reducing end to release D-glucose. *P. parasitica* contains proteins in five CAZyme families with modules associated with the degradation of starch. The families are GH13 [141], GH31 [18], CBM20 [142], CBM25 [92] and CBM47 [78]. These families have been included in the current study because most contain proteins that are predicted to be secreted (Additional file 2: Table S2) and may act on substrates other than starch. For example, α-mannosidases from the GH31 family could be involved maturation of glycoproteins [143].

Two *P. parasitica* GH13 proteins were identified. One contains the α-amylase catalytic domain, an SP and an N-terminal starch-binding CBM25 domain. A CBM25 domain was also found in one of the ten GH31 *P. parasitica* proteins [PPTG_01216]. The GH31 proteins could be divided into five subfamilies based on sequence homologies. Excluding two potentially truncated proteins, one group of three share 68% amino acid identity [PPTG_01216, 01217^CJ01A1^ and 09366^CJ01A1^], PPTG_03687 and 10577 share 70% identity, another two have 87% amino acid identity [PPTG_02261 and 07818] and one protein shared only limited homology [PPTG_12379]. Nine of the GH31 proteins are predicted to be secreted and one of these [PPTG_12379] has a predicted TMD at the C-terminus. All proteins aligned with different groups of α-glucosidases identified by BLASTp searches of NCBI, indicating they probably act in the degradation of starch. However they all shared some residues (12-18%) with a characterised α-xylosidase from *Cellvibrio japonicus* [144], leaving open the possibility that some of the *P. parasitica* GH31 family may act on terminal α-linked xylose residues of xyloglucans (Figure 2).

The final protein which may be involved in starch degradation contains both a CBM20 and a fucose-binding CBM47 module [PPTG_20189]. This is a CAZyme module combination that has not been previously described in any other organism. Initially, five CBM20/CBM47 proteins were identified by the dbCAN analysis with E-values between 7.50e-05 and 6.50e-19. However, BLAST analysis showed that two of the five have more homology to regulator-chromosome condensation proteins than to known CAZymes. The remaining three proteins [PPTG_02680, 08934 and 18605], contain other domains not associated with CAZymes. PPTG_08934, for example, contains a discoidin domain found in an adhesion protein from *Dictyostelium discoideum* [145].

### Other possible CWDEs

The *P. parasitica* genome contains six proteins in the GH32 family which includes enzymes with invertase, exo-inulinase, levanase and fructan exohydrolase activity [146]. The *P. parasitica* GH32 proteins were almost identical to *P. infestans* invertases characterised by Judelson and coworkers [147]. Two *P. parasitica* α,α-trehalases in the GH37 family both contain an SP, suggesting that they act on an extracellular source of trehalose (α-D-glucopyranosyl-α-D-glucopyranoside) to release glucose.

Two proteins containing CBM38 or CBM57 modules were identified. Few fungi contain CBM38 proteins and both CBM38 and CBM57 modules are poorly characterised [43]. Two *P. parasitica* proteins had CBM40 modules. CBM40 proteins have been found in a few fungi, but in bacteria this module has been found in sialidases [78].

### Comparison of the complement of CWDEs in *P. parasitica*with that in *P. infestans*

As a comparative exercise, we have applied our bioinformatic strategy for the identification of CWDEs to the *P. infestans* genome [148]. A summary of the numbers of carbohydrate-active proteins identified in *P. parasitica* and *P. infestans* in the current study is presented in Table 4. CAZyme families and CAZyme modules were initially identified using dbCAN, BLAST, keyword and domain searches (Additional files 3 and 4). Subsequent detailed bioinformatic analysis of individual proteins revealed that some CAZymes were cytoplasmic proteins or had alternative enzyme activities and were thus not CWDEs. Removal of these proteins from the pool of candidate CWDEs meant that the number of CWDE families is less than the number of CAZyme families in each class of enzymes. Our data show that the numbers of CWDE families and CWDE proteins in each CAZyme class are remarkably similar in *P. parasitica* and *P. infestans*. Comparison of our data with those from three other recent *P. infestans* studies [29–31, 149, 150] exemplifies the variation in CAZyme annotation achieved following different approaches. The time at which the analysis is done is also an important factor. For example, the AA class of CAZymes has only relatively recently been included within the CAZy database [21] and this explains the absence of data for AA families in the earlier *P. infestans* studies. The time at which the analysis is conducted also affects the results because of continuing changes in the annotation of the genome sequence data. In terms of the numbers of CBM, CE, GH, GT and PL CAZyme families, the numbers in the four studies are quite similar.

**Table 4:** **A summary of the numbers of carbohydrate-active proteins identified in** ***P. parasitica*** **and** ***P. infestans*** **in the current study and comparison of these data with those from three previous studies of** ***P. infestans***

	***P. parasitica***(current study)				***P. infestans***(current study)				***P. infestans***[31]		***P. infestans***[29]		***P. infestans***[149]	
CAZymes	CAZyme families	CAZyme modules	CWDE families	CWDE proteins	CAZyme families	CAZyme modules	CWDE families	CWDE proteins	CAZyme families	CAZyme modules	CAZyme families	CAZyme proteins	CAZyme families	CAZyme proteins
CBM	17	85	14	48	16	74	12	53	12	45	na	na	na	19
AA	8	43	4	13	8	42	4	13	na	na	na	na	na	na
CE	12	113	8	46	12	90	8	38	12	81	8	49	na	47
GH	37	293	34	280	36	275	35	265	34	261	34	244	na	216
GT	29	169	na	na	30	158	na	na	27	142	22	83	na	64
PL	4	47	3	44	5	67	3	54	3	57	3	59	na	59

na: data not available.

In the current study, CAZyme families and CAZyme modules were initially identified using dbCAN, BLAST, keyword and domain searches. Subsequent in-depth bioinformatic analyses of individual genes indicated that some CAZymes were unlikely to be true CWDEs, leading to a lower number of CWDE families than CAZyme families. Our data show that the numbers of CWDE families and CWDE proteins in each CAZyme class are remarkably similar in *P. parasitica* and *P. infestans*. Comparison of our *P. infestans* data with those from the other *P. infestans* studies exemplify the variation in CAZyme annotation achieved following different approaches and conducted at different times.

## Conclusions

Searches of the *P. parasitica* genome using dbCAN and BLAST identified 431 genes that potentially encode CWDEs. The 431 proteins contain 65 different CAZyme modules. Together, this cohort of 431 CWDEs has the capacity to provide *P. parasitica* with the ability to degrade all major plant cell wall components. In the study reported in the present paper, each of the 431 CWDE candidates was subjected to an in-depth bioinformatic analysis, with the goal of determining its enzymatic activity and possible substrate(s). Despite the difficulties in interpreting function from primary protein sequence, this individualized attention has not only highlighted annotation errors that are being perpetuated in successive studies but has also allowed the likely enzyme activity and targeted substrate to be deduced for many *P. parasitica* CWDEs.

In order to function in plant cell wall degradation, pathogen enzymes must be secreted and move through the pathogen cell wall to gain access to the plant cell wall. Most (337, 78%) of the 431 putative *P. parasitica* CWDE had a classical secretion SP at their N-terminus. A further 67 (16%) had sequences associated with non-classical protein secretion. Only 23 (5%) proteins, for which full-length sequence data were available, lacked any form of secretion sequence. Although initially controversial, the secretion of eukaryotic and prokaryotic proteins lacking a classical signal peptide is now well documented [49, 151]. To date, no simple motif associated with non-classical secretion has been found but together a set of about six features of the amino acid sequence allow prediction of non-classical secretion [49]. Most non-classical secretory proteins are constitutively secreted but a number of mammalian proteins that undergo regulated secretion have also been shown to follow a non-classical pathway [152]. Information from studies of the role of non-classical secretion of CWDEs is not yet available.

One hundred of the proteins with, plus an additional one without, secretion sequences, contained a TMD or a GPI anchor. While on the one hand, possession of TMDs or GPI anchors may anchor the protein to the *P. parasitica* plasma membrane, recent evidence indicates that proteins with these motifs can be released from the membrane [153, 154]. In some cases, modification of the GPI anchor results in binding of the protein to polysaccharides [155]. Biotrophic pathogens are known to have significantly fewer CWDEs than hemibiotrophs and necrotrophs, but there is little understanding of the protein attributes that might limit the movement of CWDEs and hence the degree of tissue maceration. It is known that the pH of the wall and isoelectric point of CWDEs will affect their mobility [156]. Perhaps modification of GPI anchors on CWDEs might be another means to regulate their movement within the plant cell wall.

The majority (90%) of *P. parasitica* CWDEs contain a single CAZyme module. Only 13 *P. parasitica* CWDEs have modules of different types and 37 have multiple copies of the same module. This situation is in contrast to that in fungal phytopathogens where, for example, proteins that contain a CBM module usually also include a catalytic module [22, 78, 92]. The significance of the presence of so few proteins containing both CBM and catalytic CAZyme modules in terms of *P. parasitica* pathogenicity remains to be determined, however, a CBM1 gene [PPTG_06045] is one of the most highly expressed genes during the infection of a susceptible host plant (LM Blackman, P Torreña, DP Cullerne, J Taylor and AR Hardham, unpublished observations).

Previous comparative studies of CWDEs in phytopathogenic fungi have shown that the complement of enzymes in terms of the CAZyme families represented and the numbers of proteins within a particular family are often very different in different organisms. The results of our characterization of the CWDE complement in *P. parasitica* add further data to extend this observation, and highlight some especially interesting differences between *P. parasitica*, or the Oomycetes in general, and fungal phytopathogens. The CBM CAZyme class is a case in point. In *P. parasitica*, as in *P. ramorum* and *P. sojae*, of the total of 55-61 CBM proteins, about 25% belong to CBM1 and 25% to the CBM63 families, members of which bind to cellulose. Within hemibiotrophic and necrotrophic fungal species, apart from CBMs that target chitin, CBM1 is consistently the largest CBM family, often containing 25-50% of the total non-chitin-directed CBMs [30, 31, 70, 71]. In contrast to the situation in *Phytophthora* species, there are usually only 1-3 CBM63 proteins in these fungi.

Another striking difference between *P. parasitica* and fungal phytopathogens is presented by the complements of enzymes that degrade pectin. The degradation of pectin is thought to be the first step in a cascade of CWDE activity [70, 157, 158]. Pectin degradation may increase wall porosity and expose other wall polysaccharides, thereby facilitating the action of other pathogen enzymes. A broad spectrum of pectin-degrading enzymes is believed to be required in order to degrade the diversity of pectins found in plant cell walls [159, 160]. Around 25% (108) of *P. parasitica* CWDEs are directed solely towards pectin degradation and this number increases to 50% (208) if proteins in families with multiple targets are included. Of the pectin-directed enzymes in *P. parasitica*, 44 are PLs and 35 are CEs. Similar numbers occur in other *Phytophthora* species [29, 150, 161]. This situation may be compared to that in the fungi which typically have fewer than 20 PLs and 14 CEs [69–71, 162]. In fungal pathogens, the pectin degrading enzymes are predominantly GH proteins [69–71, 163, 164].

A third interesting comparison between Oomycete and fungal pathogens concerns the numbers of CWDEs directed towards the degradation of β-1,3-glucans. The deposition of β-1,3-glucans, in the form of callose, is an integral part of the basal plant defence response [165–167], and thus an ability to degrade callose could be an important attribute for successful infection. The *P. parasitica* genome contains 14 GH72 and 16 GH81 proteins that are thought to specifically degrade β-1,3-glucans. Fungal phytopathogens studied to date, on the other hand, have 1-8 GH72 and 0-3 GH81 proteins [69–71, 162]. These fungi may have up to about 4 or 6 β-1,3-glucanases in GH55 and GH64 families, respectively. *Phytophthora* cell walls themselves contain β-1,3-glucans [77, 129], so perhaps some of the *P. parasitica* β-1,3-glucanases are involved in pathogen wall modifications. However, it is still pertinent to ask if the larger numbers of β-1,3-glucanases in the Oomycetes mean that these organisms are better equipped to degrade the callose that is rapidly deposited in wall appositions at the infection site? An answer to this, and many other questions on CWDE function, requires further information, in particular that arising from transcriptome and protein localization studies.

## Electronic supplementary material

Additional file 1: **Putative CAZyme modules identified by dbCAN analysis of predicted** ***P. parasitica*** **INRA-310 (V2) proteins.** This output gives the E-value of the comparison of the dbCAN conserved domain determined by Hidden Markov Models and the signature domain of the queried protein. Columns four and five show the start and end of the aligned region within the conserved domain. Column six shows the fraction of the conserved domain covered by the *P. parasitica* signature domain. Columns seven and eight show the location of the signature domain within the *P. parasitica* protein. Five proteins from alternatively spliced transcripts (T1) that contained CAZyme modules were omitted from the analysis as these transcript variations did not result in different proteins. Where a protein was different in the most recent version of the annotated genome [46], the newer entry was used and these are indicated by (V3). Proteins that appeared to be incorrectly annotated as seen by missing 5’ or 3’ sequence data or to have incorrectly placed introns (as indicated by comparisons with EST data or genomic sequence data from another *P. parasitica* isolate), were manually curated and re-analyzed. Notes on proteins that appeared to be CAZymes but had greater homology to other proteins are included in the Comments column. Any protein with a dbCAN E-value > e-10 or an aligned coverage fraction <0.5 were further scrutinised to ensure that no putative CWDEs were incorrectly identified. Gray shading indicates that the predicted protein contains more than one CAZyme module of the same type. Green shading indicates that the predicted protein contains CAZyme modules of different types. TMD indicates a transmembrane domain. (XLS 132 KB)

Additional file 2: **Details of putative** ***P. parasitica*** **INRA-310 (V2) CWDEs and their potential substrates.** Truncated proteins and the *P. parasitica* strains used for analysis of specific proteins are indicated (P1569, P1976, P10297 and CJ01A1). HG = homogalacturonan, RGI = rhamnogalacturonan I, AGP = arabinogalactan proteins, GA = galacturonic acid. V3 = protein from *P. parasitica* INRA-310 (V3) used for analysis. The number of CAZyme modules and their E-values from dbCAN analysis or other features such as a Pfam domain are shown. SP = secretion signal determined by SignalP; “SP” = secretion signal if alternative nearby start codon is used; NN scores ≥ 0.5 = non-classical secretion; TMD = transmembrane domain, the location of which is indicated by N or C; GPI = glycosylphosphatidylinositol-anchored, ? = complete analysis was not possible due to truncated proteins in all *P. parasitica* strains and it was not possible to determine the likely substrate. (XLS 94 KB)

Additional file 3: **Putative CAZyme modules identified by dbCAN analysis of predicted** ***P. infestans*** **proteins.** The output gives the E-value of the comparison of the dbCAN conserved domain determined by Hidden Markov Models and the signature domain of the queried protein. Columns four and five show the start and end of the aligned region within the conserved domain. Column six shows the fraction of the conserved domain covered by the *P. infestans* signature domain. Columns seven and eight show the location of the signature domain within the *P. infestans* protein. Gray shading indicates that the predicted protein contains more than one CAZyme module of the same type. Green shading indicates that he predicted protein contains CAZyme modules of different types. (XLS 120 KB)

Additional file 4: **Details of putative** ***P. infestans*** **CWDEs.** The number of CAZyme modules and their E-values from dbCAN analysis or other features such as a Pfam domain are shown. Truncated proteins are indicated. SP = secretion signal determined by SignalP; “SP” = secretion signal if alternative nearby start codon is used; NN scores ≥ 0.5 = non-classical secretion; TMD = transmembrane domain, the location of which is indicated by N or C; GPI = glycosylphosphatidylinositol-anchored. (XLS 73 KB)

## Abbreviations

AA: Auxiliary activities
AGP: Arabinogalactan protein
CAT: CAZymes Analysis Toolkit
CAZyme: Carbohydrate-Active enzyme
CBM: Carbohydrate binding module
CE: Carbohydrate esterase
CWDE: Cell wall degrading enzyme
dbCAN: Carbohydrate-active enzyme Annotation
GH: Glycoside hydrolase
GPI: Glycosylphosphatidyl inositol
GT: Glycosyl transferase
HG: Homogalacturonan
NCBI: National Center for Biotechnology Information
PGA: Polygalacturonic acid
PL: Polysaccharide lyase
PPTG: *Phytophthora parasitica* INRA-310 (V2) protein
PITG: *Phytophthora infestans* protein
RGI: Rhamnogalacturonan I
RGII: Rhamnogalacturonan II
SP: Secretion signal
TMD: Transmembrane domain.
